# Prevalence of Malaria and Associated Factors Among Patients Attending Finote Selam General Hospital, West Gojjam, Northwest Ethiopia

**DOI:** 10.1155/ghe3/6818877

**Published:** 2026-11-04

**Authors:** Abrhi Tsehay, Sissay Menkir, Habtamu Mekonnen

**Affiliations:** ^1^ Department of Biology College of Science Bahir Dar University Bahir Dar P.O. Box 79 Ethiopia bdu.edu.et

**Keywords:** associated factors, hospital-based cross-sectional study, insecticide-treated nets, malaria prevalence, *Plasmodium* species

## Abstract

**Background:**

Malaria, caused by *Plasmodium* parasites, is a significant health issue in Ethiopia, including the current study area. However, its epidemiology and associated factors are not well understood. This study aimed to determine the prevalence of malaria and associated factors among patients attending Finote Selam General Hospital, northwest Ethiopia.

**Methods:**

A hospital‐based cross‐sectional study was conducted at Finote Selam General Hospital in April–May 2022. A structured questionnaire was used to collect sociodemographic and other explanatory variables from 424 randomly selected participants. Descriptive statistics were used to calculate malaria prevalence. Pearson’s chi‐square test examined the relationship between malaria cases and independent factors. Bivariate logistic regression identified variables with *p* values < 0.25, which were then included in multivariate logistic regression. Differences with *p* values < 0.05 were considered statistically significant.

**Results:**

Out of 424 blood samples, 93 (21.9%) were malaria‐positive. Among these cases, 69.9% were *P*. *falciparum*, 23.7% were *P*. *vivax*, and 6.5% were mixed infections. Multivariate logistic regression revealed significant associated factors: age group (*p* = 0.001), participant’s residence (*p* < 0.001), occupation (*p* < 0.001), and family income (*p* = 0.002), knowledge of malaria prevention (*p* = 0.036), environmental sanitation (*p* = 0.032), stagnant water near home (*p* < 0.001), and ITN usage (*p* = 0.001).

**Conclusions:**

Malaria remains an important public health concern in the study area. Several demographic, environmental, and behavioral factors were significantly associated with malaria infection. Strengthening existing malaria prevention and control measures and conducting further entomological and longitudinal studies are recommended.

## 1. Introduction

Malaria is an infectious disease caused by protozoan parasites belonging to the genus *Plasmodium*. The major *Plasmodium* species known to infect humans include *P*. *falciparum*, *P. vivax*, *P. ovale*, *P. malariae*, and *P. knowlesi*. Moreover, zoonotic malaria parasites, such as *P*. *cynomolgi*, have recently been recognized as emerging causes of human malaria infection, expanding the diversity of *Plasmodium* species of public health importance [1]. The female *Anopheles* mosquito is a vector that can carry the parasite and transmit the disease from an infected individual to a healthy one. The vector favors a high‐temperature climate for its reproduction. The parasite also requires a warm environment to complete its growth cycle in the vector before reaching the stage at which it is ready to be transmitted to humans [2]. Thus, the disease is commonly found in the warmest countries that are closer to the equator, including tropical and subtropical countries.

Malaria is one of the major diseases that cause significant mortality and morbidity worldwide. In tropical and subtropical regions of the world, malaria causes considerable impact in the medical, social, and economic sectors [3]. According to Kolawole *et al.* [4], the estimated global malaria cases in 2020 were 241 million, with 6,27,000 malaria deaths, with the African continent accounting for 95% of malaria cases and 96% of malaria deaths. David *et al.* [5] report that one‐half of the world’s population is still at risk of malaria. In African countries, particularly sub‐Saharan Africa (SSA), including Ethiopia, malaria is more common, and more than 50% of the population is at risk.

As Ethiopia is one of the countries in SSA, malaria remains one of the major public health problems. It is estimated that 68% of the country’s land is malarious, with about 60% of the total population at risk of malaria [6]. Accordingly, the most dominant malaria parasites in the country are *P. falciparum* and *P. vivax,* which account for 60% and 40% of malaria cases, respectively. Although the burden of malaria in Ethiopia has been reduced remarkably through public health interventions designed during the Millennium Development Goals, there was an increase in the number of malaria cases in 2019/2020. This happened because of the COVID‐19 pandemic, security issues, the existence of internally displaced populations (IDPs) in various parts of the country, poor performance on Larval Source Management (LSM) and net utilization, and the low commitment of leaders [7]. The most malarious regions in Ethiopia are the Southern Nations, Nationalities, and Peoples’ Region (SNNPR), Oromia Regional State, and Amhara National Regional State (ANRS) [8].

The ANRS is Ethiopia’s third‐most malarious region. Approximately 80% of the region’s land is malarious, and about 68% of the population is at risk of malaria [7]. According to the Yenew and Mulatu [9] report, the highest annual average incidence rate of malaria was found in North Gondar (59.7), Awi (54.3), South Gondar (27.5), and West Gojjam (26.3) per 1000 population. Accordingly, the West Gojjam Zone is one of the malaria hotspot areas in ANRS. Moreover, among all the distinct districts in West Gojjam Zone, the annual incidence of malaria was very high in Jabi Tenan district in 2015/16 [10]. Malaria is still a major public health challenge and causes severe illness and death in Finote Selam town, since it belongs to a malaria hotspot zone surrounded by Jabi Tenan district. In addition, as with most developing‐country towns, the current study area is characterized by poor housing, a lack of proper sanitation, poor surface water drainage, inadequate health services, and widespread economic disparity, all of which contribute to the path of urban malaria transmission [11]. Therefore, malaria is still a major public health problem in many parts of the country, including the present study area. Although the burden of the disease is very high in the current study area, little is known about its current epidemiology and its influencing factors.

A wide range of malaria‐related factors that are critical to public health actions were not well addressed in Ethiopia, including the current study site. There was no other recent research report about the prevalence of malaria and its associated factors in Finote Selam town. Therefore, this study aimed to assess the prevalence of malaria and associated factors among patients attending Finote Selam General Hospital (FSGH), West Gojjam, northwest Ethiopia.

## 2. Material and Methods

### 2.1. Description of the Study Area

Finote Selam is the capital town of the West Gojjam Zone in the ANRS, in northwestern Ethiopia. It is located 387 km from Addis Ababa and 176 km from Bahir Dar. It is surrounded by Jabi Tehnan. The town has an elevation of 1917 m above sea level, with a longitude and latitude of 10° 42′ N and 37° 16′ E. The mean annual temperature and precipitation are 17.8°C and 1450.3 mm, respectively. According to the 2007 census report, the town has a total population of 25,913 (https://dbpedia.org/page/Finote_Selam). Finote Selam town and the surrounding areas have good potential for irrigation agriculture, with an actual surface irrigation potential of 47 km [12]. The town has 20 health‐related institutions, i.e., it has one general hospital, one health center, two health posts, and 10 private clinics (Finote Selam General Hospital Health Management and Information System Office, 2022). The hospital was built in 1964 with the contribution of local people and the private fund “Swedish Aid to Leprous Children in Ethiopia.” Now the hospital is one of the large hospitals found in ANRS.

### 2.2. Study Design and Period

A hospital‐based cross‐sectional study was conducted at FSGH, northwest Ethiopia, from April to May 2022.

### 2.3. Study Population and Source of the Population

All patients who visited the FSGH during the study period were considered the source population for this study. Those patients suspected of having malaria who visited the FSGH during the study period were considered the study population.

### 2.4. Inclusion and Exclusion criteria

All malaria‐suspected patients who attended the FSGH during the data collection time and provided informed written consent were recruited. All eligible patients of all ages and both sexes were included in the present study. Patients who did not provide informed written consent and those individuals who had received antimalarial treatment during the 3 weeks prior to enrolment were excluded from the study.

### 2.5. Variables

The dependent variable was malaria positivity (presence of malaria represented by “Pos” and absence of malaria by “Neg”) among patients attending the FSGH, which was determined using microscopic diagnosis of blood samples from study participants. The independent variables of this study include age, gender, occupation, family size, place of residence, wealth level, education level, the main material of the roof, insecticidal bed net utilization, indoor residual spraying (IRS), outdoor sleeping, knowledge of malaria, presence of stagnant water, wearing of long cloth at night, previous malaria parasite infection, and environmental sanitation status.

### 2.6. Sample Size Determination and Sampling Technique

#### 2.6.1. Sample Size Determination

The required sample size was determined by using a single proportion formula [13]. *n* = *Z* ^2^ *P*(1 − *P*)/*d* ^2^. A prevalence estimate of 50% (*p* = 0.5) was used because there was no previous study reporting a comparable malaria prevalence among malaria‐suspected patients attending FSGH. Using *p* = 0.5 provides the maximum sample size and ensures adequate precision of the prevalence estimate. The calculation was based on a 95% confidence interval (*Z* = 1.96) and 5% marginal error (*d* = 0.05). The calculated sample size was 384.16, and after adding a 10% allowance for nonresponse, the final sample size was 424 participants.

#### 2.6.2. Sampling Technique

The study participants were selected using a systematic random sampling technique. According to the hospital registration records, an average of 848 malaria‐suspected patients attended the outpatient department during the 12 months preceding the study. The sampling interval (*K*) was calculated by dividing the average number of malaria‐suspected patients by the required sample size (424), as follows:

(1) K=Nn=848424=2.

Therefore, every second eligible malaria‐suspected patient was selected for inclusion in the study. The first participant was selected randomly using a lottery method from the first two eligible patients. Thereafter, every second eligible patient was recruited until the required sample size of 424 participants was achieved.

### 2.7. Data Collection

#### 2.7.1. Questionnaire Survey

Structured questionnaires were prepared to collect sociodemographic variables and other possible associated factors for malaria. Family monthly income was collected using predefined categories in the questionnaire and classified as low (< 1000 Ethiopian Birr/month), medium (1000–2000 Ethiopian Birr/month), and high (> 2000 Ethiopian Birr/month). These categories were used in the subsequent statistical analyses. Trained data collectors who had at least a diploma in health sciences were recruited. In the case of patients who were children, their parents or guardians participated in filling out the questionnaires. Face‐to‐face interviews based on questionnaires were also implemented for those participants who were unable to read and fill out the questionnaires. The questionnaires were first pretested using a few respondents (15 individuals) who were outside the study population.

#### 2.7.2. Laboratory Diagnosis

Microscopic diagnosis through thin and thick blood film preparations and examinations was taken as the gold standard for the detection of malaria parasites [14]. Capillary blood was obtained by pricking the patient’s finger with a sterile lancet, and the required blood sample was collected by a micropipette. Thick and thin blood smears were prepared for the purpose of checking the presence of *Plasmodium* parasites and species identification based on WHO guidelines. Then, both blood smears were stained with a Giemsa stain solution for 10 min. Each blood smear had been examined by a microscope under the objective of oil immersion. Finally, the presence or absence of *Plasmodium* species was identified in the thick smear, and each *Plasmodium* species was identified in the thin blood smear.

### 2.8. Data Quality Control

The data collectors were trained, and they were blind to the malaria status of the enrolled individuals. All negative and positive test slides were stored in a slide box in the hospital. Three‐quarters of the samples were randomly selected and cross‐checked by a senior and qualified laboratory technician in the FSGH who was blind to the first result. The filled‐out questionnaires were evaluated by the principal investigator every day for completeness and consistency of the data. The principal investigator also counted and documented the number of confirmed and suspected cases at the end of each data collection before forwarding the data for analysis.

### 2.9. Data Analysis

Data were entered and analyzed using SPSS 26. The overall magnitude of malaria infection was analyzed using descriptive statistics through frequencies. Pearson’s chi‐square (*x* ^2^) test was used to determine the association between the cases of malaria and its independent factors. A bivariate logistic regression analysis was used to determine the strength of the association between independent and dependent variables. Those variables that had a *p* value less than 0.25 in bivariate logistic regression were selected for multivariate logistic regression analysis using a backward elimination method [15]. The differences were considered statistically significant when the calculated *p* value was less than 0.05.

## 3. Results

### 3.1. Sociodemographic Characteristics of the Study Participants

Blood samples and filled‐out questionnaires were collected from 424 randomly selected study participants. The number of female and male study participants was 227 (53.5%) and 197 (46.5%), respectively (Table 1). The age of the participants ranged from 2 days to 87 years, with a mean age of 24.64 ± 18.26. The majority of the study participants were in the age group of ≥ 36 years (35.1%). Of the total study participants, 164 (38.7%) were farmers and 123 (29%) were illiterate. The majority of the study participants were urban residents. The average family size per household was 3.17, with a range of 1–8 individuals per household. Most of the study participants (87.5%) had a house roof made of mud (Table 1).

**TABLE 1:** Sociodemographic characteristics of the study participants, Finote Selam General Hospital, Northwest Ethiopia, April to May 2022 (*n* = 424).

Variables	Categories	Frequency	Percentage (%)
Gender	Male	197	46.5
	Female	227	53.5
	Total	424	100.0

Age	≤ 5	37	8.7
	6–17	94	22.2
	18–25	77	18.2
	26–35	67	15.8
	≥ 36	149	35.1
	Total	424	100

Residence	Urban	262	61.8
	Rural	162	38.2
	Total	424	100

Education	Illiterate	123	29.0
	Literate	301	71.0
	Total	424	100.0

Occupation	Farmer	164	38.7
	Government Employer	72	17.0
	Merchant	35	8.3
	Daily laborer	43	10.1
	Student	8	1.9
	Other	102	24.0
	Total	424	100.0

Monthly Income (In Birr)	Low	111	26.2
	Medium	205	48.3
	High	108	25.5
	Total	424	100.0

Roof	Mud	371	87.5
	Tin sheet	53	12.5
	Total	424	100.0

Family	≤ 4	298	70.3
	≥ 5	126	29.7
	Total	424	100.0

*Note:* ETB, Ethiopian Birr; family income categories: low (< 1000 ETB/month), medium (1000–2000 ETB/month), and high (> 2000 ETB/month).

### 3.2. Prevalence of Malaria in Relation to Sociodemographic Characteristics

Out of a total of 424 study participants, the prevalence of malaria was 21.9%. Malaria was found in 24.9% of male participants and 19.4% of female participants (Table 2). Based on the age‐specific prevalence, the highest prevalence of malaria was observed among the age group of under 5 years (≤ 5), at 29.7%, whereas the lowest prevalence of malaria was recorded among the age group greater than or equal to 36 years (16.8%). Malaria parasite infection was more common in daily laborers (46.5%), followed by farmers (31.7%), than in other occupations. Malaria prevalence was found to be greater in rural residents (36.4%) compared to urban residents (13%). Illiterates were found to have a higher rate of malaria parasite infections (37.4%) than literates (15.6%). Malaria was more common in people who lived with more than or equal to five individuals per household than in those who lived with less than or equal to four individuals per household. In addition, the prevalence of malaria parasite infection was relatively higher among individuals who lived in low family income levels (51.4%) than those who lived in high income levels (6.5%) and medium family income levels (14.1%) (Table 2).

**TABLE 2:** Prevalence of malaria in relation to sociodemographic characteristics of the study participants in Finote Selam General Hospital, Northwest Ethiopia, April to May, 2022.

Variables	Categories	No. of examined	No. of positive	Prevalence (%)	*X* ^2^	*p* value
Sex	Male	197	49	24.9	1.86	0.173
	Female	227	44	19.4		
	Total	424	93	21.9		

Age group (years)	≤ 5	37	11	29.7	4.847	0.303
	6–17	94	20	21.3		
	18–25	77	20	26.0		
	26–35	67	17	25.3		
	≥ 36	149	25	16.8		
	Total	424	93	21.9		

Residence	Rural	162	59	36.4	32.13	*p* < 0.001
	Urban	262	34	13.0		
	Total	424	93	21.9		

Educational status	Illiterate	123	46	37.4	24.2	*p* < 0.001
	Literate	301	47	15.6		
	Total	424	93	21.9		

Occupation	Farmer	164	52	31.7	44.6	*p* < 0.001
	Government employer	72	6	8.3		
	Merchant	35	4	11.4		
	Daily laborer	43	20	46.5		
	Student	8	2	25		
	Other	102	9	8.8		
	Total	424	93	21.9		

Family income level	Low	111	57	51.4	78.4	*p* < 0.001
	Medium	205	29	14.1		
	High	108	7	6.5		
	Total	424	93	21.9		

House roof type	Mud	371	87	23.5	3.98	0.046
	Tin sheet	53	6	11.3		
	Total	424	93	21.9		

No. of individuals per household	≤ 4	298	60	20.1	1.404	0.168
	≥ 5	126	33	26.2		
	Total	424	93	21.9		

*Note:* All *p* values were reported to three decimal places, and values less than 0.001 were presented as *p* < 0.001.

### 3.3. Prevalence of Malaria with Respect to *Plasmodium* Species

From the total malaria cases, the prevalence of *P. falciparum*, *P. vivax*, and their mixed infections was 15.3%, 5.2%, and 1.4%, respectively. Accordingly, *P. falciparum* was the most prevalent species over others, and most cases of malaria among the study participants were due to this species of *Plasmodium* (Table 3). Regarding the *Plasmodium* species with respect to the gender of the participant, the number of cases of malaria due to *P. vivax* infection was higher in females (63.6%) than males, whereas males were found to have the highest rates of mixed and *P. falciparum* infections. However, this difference was not statistically significant (*p* = 0.15).

**TABLE 3:** The prevalence of *Plasmodium* species with respect to age and gender among patients attending Finote Selam General Hospital, Northwest Ethiopia, April to May 2022.

Variables	Categories	*P. falciparum n* (%)	*P. vivax n* (%)	Mixed *n* (%)	Total *n* (%)	*X* ^2^	*p* value
Gender	Male	37 (18.8)	8 (4.1)	4 (2.0)	49 (24.9)	5.15	0.15
	Female	28 (12.3)	14 (6.2)	2 (0.9)	44 (19.4)		
	Total	65 (15.3)	22 (5.2)	6 (1.4)	93 (21.9)		

Age	≤ 5	8 (21.6)	1 (2.7)	2 (5.4)	11 (29.7)	11.4	0.49
	6–17	13 (13.8)	6 (6.4)	1 (1.1)	20 (21.3)		
	18–25	15 (19.5)	4 (5.2)	1 (1.3)	20 (26.0)		
	26–35	13 (19.4)	3 (4.5)	1 (1.5)	17 (25.3)		
	≥ 36	16 (10.7)	8 (5.4)	1 (0.7)	25 (16.8)		
	Total	65 (15.3)	22 (5.2)	6 (1.4)	93 (21.9)		

*Note:* Percentages were calculated using the total number of participants examined within each age or gender category as the denominator. Thus, the percentages represent the prevalence of each *Plasmodium* species within the corresponding subgroup. *n*, number of malaria‐positive participants. *X* ^2^, chi‐square statistic.

The prevalence of *P. falciparum* infection was relatively higher in the age group ≤ 5 than in other age groups, whereas *P*. *vivax* infection was slightly higher in the age group between 6 and 17. However, this difference was not statistically significant (*p* = 0.49) (Table 3).

### 3.4. Bivariate and Multivariate Logistic Regression of Associated Factors of Malaria

Among the 16 independent factors, 11 variables showed a statistically significant association (*p* ≤ 0.05) in the bivariate analysis, while variables with *p* < 0.25 were considered for entry into the multivariable logistic regression model. These variables were residence of the participant, educational status, occupation, family income, knowledge on malaria prevention and control, experience of outdoor sleeping or working at night, hole in the wall of the house, presence of stagnant water around home, regular wearing of long clothes at night, previous infection, and ITN use status. On the other hand, the remaining five variables (gender, age, number of individuals per household, cleaning the environment regularly, and roof type of the house) did not show a significant association with malaria parasite infections (*p* > 0.05) (Table 4). In addition, none of the respondents used IRS in their houses during the previous 6 months.

**TABLE 4:** Bivariate and multivariate logistic regression analysis of associated factors for malaria parasite infection, Finote Selam General Hospital, Northwest Ethiopia, April to May 2022.

Variables	Categories	Malaria		Bivariate analysis		Multivariate analysis	
		Pos (*n*)	Neg (*n*)	COR (95% CI)	*p* value	AOR (95% CI)	*p* value
Gender	Male	49	148	1.38 (0.87, 2.18)	0.174		
	Female	44	183	1			

Age	≤ 5	11	26	2.09	0.078	5.93 (2.02, 17.45)	0.001
	6–17	20	74	1.34	0.380	1.63 (0.69, 3.83)	0.266
	18–25	20	57	1.74	0.103	1.45 (0.60, 3.53)	0.412
	26–35	17	50	1.69	0.142	0.95 (0.38, 2.35)	0.908
	≥ 36	25	124	1		1	

Residence	Rural	59	103	3.84 (2.37, 6.22)	*p* < 0.001	5.14 (2.39, 11.09)	*p* < 0.001
	Urban	34	228	1		1	

Educational status	Illiterate	46	77	3.23 (2.00, 5.22)			
	Literate	47	254	1			

Occupation	Farmer	52	112	4.80 (2.25, 10.25)	*p* < 0.001	2.08 (0.78, 5.53)	0.143
	Government employer	6	66	0.94 (0.32, 2.77)	0.910	2.49 (0.62, 9.93)	0.198
	Merchant	4	31	1.33 (0.38, 4.64)	0.651	2.03 (0.47, 8.80)	0.346
	Daily laborer	20	23	8.99 (3.62, 22.31)	*p* < 0.001	9.41 (2.91, 30.49)	*p* < 0.001
	Student	2	6	3.44 (0.60, 19.633)	0.164	3.72 (0.40, 35.02)	0.251
	Others	9	93	1		1	

Family income level	Low	57	54	15.23 (6.50, 35.69)	*p* < 0.001	5.95 (1.93,18.30)	0.002
	Medium	29	176	2.38 (1.01,5.62)	0.049	1.20 (0.39, 3.70)	0.748
	High	7	101	1		1	

Roof type of the house	Mud	87	284	2.40 (0.99, 5.80)	0.052		
	Tin sheet	6	47	1			

Family size per household	≤ 4	60	238	1			
	≥ 5	33	93	1.41 (0.86, 2.29)	0.170		

Knowledge on malaria	Yes	49	256	1		1	
	No	44	75	3.07 (1.89, 4.96)	*p* < 0.001	1.98 (1.05, 3.74)	0.036

Outdoor activities	Yes	43	70	3.21 (1.97, 5.21)	*p* < 0.001		
	No	50	261	1			

Hole in the wall	Yes	33	77	1.81 (1.11, 2.98)	0.018		
	No	60	254	1			

Wearing long clothes	Yes	35	195	1			
	No	58	136	2.38 (1.48, 3.81)	*p* < 0.001		

Previous infection	Yes	26	53	2.04 (1.19, 3.49)	0.010		
	No	67	278	1			

Stagnant water	Yes	49	48	6.50 (3.95, 10.93)	*p* < 0.001	4.74 (2.41, 9.32)	*p* < 0.001
	No	44	283	1		1	

Clean environment	Yes	65	260	1		1	
	No	28	71	1.58 (0.94, 2.64)	0.083	2.18 (1.07, 4.45)	0.032

Using ITN regularly	Yes	59	292	1		1	
	No	34	39	4.32 (2.52, 7.39)	*p* < 0.001	3.47 (1.65, 7.29)	0.001

*Note:* All *p* values were reported to three decimal places, and values less than 0.001 were presented as *p* < 0.001. Pos, positive; *n*, number; Neg: negative.

Abbreviations: AOR, adjusted odds ratio; CI, confidence interval; COR, crude odds ratio.

Potential malaria‐associated factors with a *p* value <0.25 in the bivariate analysis were entered into the multivariable logistic regression model, and backward elimination was applied to identify factors independently associated with malaria parasite infection [15]. After backward elimination, eight variables remained in the final model: age group, residence, occupational status, family income level, knowledge of malaria prevention and control methods, environmental sanitation, presence of stagnant water near the home, and ITN use (*p* < 0.05). Male participants had higher odds of malaria infection than female participants. However, the association was not statistically significant (AOR = 1.38, 95% CI: 0.87–2.18; *p* = 0.174). Children aged ≤ 5 years had approximately six times higher odds of malaria parasite infection than those aged ≥ 36 years (AOR = 5.93, 95% CI: 2.02–17.45, *p* = 0.001). Individuals residing in rural areas had approximately five times higher odds of malaria parasite infection than urban residents (AOR = 5.14, 95% CI: 2.39–11.09, *p* < 0.001). Daily laborers had approximately nine times higher odds of malaria parasite infection than individuals in the reference occupational category (AOR = 9.41, 95% CI: 2.90–30.49, *p* < 0.001).

The analysis also showed that the association between malaria parasite infection and the presence of a water body around the home was statistically significant, in which individuals who lived in households located near stagnant water or other water bodies had approximately 4.8 times higher odds of malaria parasite infection than those who had no stagnant water around their home (AOR = 4.74, 95% CI = 2.41–9.32).

In addition, those individuals who lived in households that had a lower income per month had approximately six times higher odds of malaria parasite infection than those who lived in households with a higher monthly income (AOR = 5.95, 95% CI: 1.93–18.30). The association was statistically significant (*p* = 0.002). Those individuals who did not know about malaria prevention and control methods had approximately two times higher odds of malaria parasite infection than those who did (AOR = 1.98, 95% CI: 1.05–3.74). The association was statistically significant (*p* = 0.036). Moreover, the likelihood of having malaria in those individuals who did not clean their environment had approximately two times higher odds of malaria parasite infection than in those individuals who cleaned their environment regularly (AOR = 2.18, 95% CI: 1.07–4.45). The association was statistically significant (*p* = 0.032).

Individuals who did not use ITN on a regular basis had approximately 3.5 times higher odds of malaria parasite infection than those who did (AOR = 3.47, 95% CI = 1.65–7.29), and the association was statistically significant (*p* = 0.001). Furthermore, individuals who had participated in night‐time outdoor activities had approximately three times higher odds of malaria parasite infection than their counterparts (COR = 3.21, 95% CI = 1.97–5.21). Moreover, individuals who did not wear long clothes had approximately 2.4 times higher odds of malaria parasite infection than those who wore long clothes regularly at night (COR = 2.38, 95% CI = 1.48–3.81). However, according to multivariable logistic regression analysis, the association between malaria parasite infection and household size, educational status, previous malaria infection, presence of holes in the wall of the house, and roof type was not statistically significant (*p* > 0.05) (Table 4).

## 4. Discussion

According to the results of this study, the prevalence of malaria among the study participants was 21.9%. This prevalence was comparable to the findings reported from other parts of Ethiopia (22.4%) [16], (21.1%) [17], (22.1%) [18], and (25%) [19]. In contrast, it was lower than the prevalence reported in previous studies (39.6%) [20], (36.1%) [21], (29%) [22], and (44%) [23], but higher than those reported in other Ethiopian studies (5%) [24], (5.6%) [25], (7.7%) [26], (4.2%) [27], and (4.5%) [28]. The observed variation in malaria prevalence across studies may be explained by differences in the ecological and epidemiological characteristics of the study area, methodological approaches, and the effectiveness of malaria control interventions. The present study was conducted in Finote Selam, located at an altitude of approximately 1917 m above sea level, with a mean annual temperature of 17.8°C and annual rainfall of about 1450 mm. In addition, the surrounding area has irrigation‐based agricultural activities that may provide suitable conditions for mosquito breeding. These ecological characteristics may influence vector abundance and malaria transmission and may have contributed to the observed malaria prevalence. However, because the present study did not include entomological assessments, including evaluation of mosquito breeding habitats or vector abundance, these environmental factors should be interpreted as possible contributors rather than direct evidence of the mechanisms underlying malaria transmission in the study area. In contrast, studies conducted in lower altitude or more highly endemic malaria areas often report higher prevalence because environmental conditions are generally more favorable for mosquito breeding and sustained malaria transmission, whereas studies conducted in higher altitude or low‐transmission settings tend to report lower prevalence. Moreover, differences in malaria diagnostic methods may have contributed to the observed variation. Studies using more sensitive diagnostic techniques, such as polymerase chain reaction (PCR), can detect low‐density and asymptomatic infections that may be missed by microscopy or rapid diagnostic tests (RDTs), resulting in higher reported prevalence. Differences in sampling techniques, study design, season of data collection, and the implementation of malaria prevention and control measures may also have influenced the observed variation among the studies [26, 29].

The current study also revealed that *P. falciparum* was the most dominant species over others, followed by *P. vivax* with a relative proportion of 69.9% and 23.7%, respectively. The remaining 6.5% of the malaria positivity rate was due to mixed infections. This finding is supported by [21]. However, the current finding is not in line with a nationwide report from Ethiopia [6]. This might be due to the fact that the national estimate of malaria parasites indicates the average distribution in the whole country, while the current study is limited to only a small part of Ethiopia, since the epidemiological distribution of *Plasmodium* species in different parts of Ethiopia is different [30].

The current study also assessed the potential associated factors for malaria parasite infection among patients attending FSGH. Malaria parasite infection was associated with gender, age group, residence, number of individuals per household, monthly family income level, occupation, roof type of the house, knowledge of malaria prevention and control, outdoor sleeping or working at night, presence of a hole in house walls, presence of stagnant water around home, environmental sanitation, wearing long clothes at night, previous malaria infection, and ITN use. However, in the multivariable logistic regression analysis, only some of these variables remained independently associated with malaria parasite infection. This finding is consistent with the report by Abebaw [31]. Male participants had higher odds of malaria infection than female participants (AOR = 1.38), but the association was not statistically significant (*p* = 0.174). This finding is consistent with the previous reports [16, 32, 33], although other studies have reported higher odds of infection among females [22]. The observed higher odds among males may be related to differences in exposure patterns, as males are often more involved in outdoor activities, including farming and overnight stays in the farm areas, which may increase contact with malaria vectors. However, since the association was not statistically significant in the present study, this finding should be interpreted with caution.

Children aged ≤ 5 years had higher odds of malaria infection compared with individuals aged ≥ 36 years, and this association was statistically significant (*p* = 0.001). This finding is consistent with previous studies [32, 34]. In contrast, other studies reported higher odds of malaria among individuals older than 15 years compared to other age groups [35, 36]. These differences may reflect variations in the study population, sample size, and sampling techniques, and local malaria epidemiology. In addition, children younger than 5 years generally have less developed immunity to malaria parasites than older individuals, which may increase their susceptibility to infection [37]. The odds of malaria among rural residents were five times higher than those among urban residents (*p* < 0.001). This finding is consistent with previous reports [25, 33, 38]. The higher odds observed among rural residents may be associated with poorer housing conditions, environmental characteristics favorable for *Anopheles* mosquito breeding, and behavioral factors that increase exposure to mosquito bites [39]. Furthermore, there was a statistically significant association between malaria parasite infection and occupation. Daily laborers had 9.4 times higher odds of malaria infection than individuals in other occupational groups (*p* < 0.001). This study was in line with previous studies [33, 40, 41]. The higher odds among daily laborers may be related to occupational mobility between areas with different malaria transmission intensities and greater participation in outdoor activities during periods of mosquito activity, which may increase exposure to infective bites.

The current finding showed that the presence of stagnant water bodies around the home was significantly associated with malaria parasite infection. Individuals living in households located near stagnant water bodies had 4.7 times higher odds of malaria infection compared with those who did not have stagnant water or nearby water bodies around their homes (*p* < 0.001). This finding is consistent with previous studies [40, 42, 43]. The observed association may be related to the potential role of stagnant water as a favorable environmental condition for malaria‐vector breeding and increased human‐vector contact. However, the present study did not assess the presence of *Anopheles* larvae, vector density, or breeding‐site productivity; therefore, the relationship between stagnant water and malaria infection should be interpreted as an epidemiological association rather than direct evidence of vector breeding. Further entomological investigations are needed to characterize malaria‐vector distribution, breeding habitats, and transmission dynamics in the study area.

Regarding family income level, individuals living in households with the lowest income had six times higher odds of malaria infection compared with those living in households with the highest income level, and the association was statistically significant (*p* = 0.002). This result agrees with other studies [38, 40, 44]. Moreover, individuals living in households with five or more members had 1.4 times higher odds of malaria infection compared with those living in smaller households; however, this association was not statistically significant (*p* = 0.17). This finding is consistent with previous reports [34, 38, 44]. The observed association may be related to increased household occupancy, which could potentially influence malaria exposure through factors such as increased human presence and related host cues that may attract mosquitoes [32]. However, this relationship should be interpreted cautiously because household‐related environmental and entomological factors were not directly assessed in the present study. Regarding the association between malaria parasite infection and the use of insecticide‐treated bed nets, individuals who did not use ITNs regularly had 3.5 times higher odds of malaria infection compared with those who did. This finding is supported by previous studies [17, 40, 44, 45].

In addition, individuals who participated in outdoor activities and slept outside at night had three times higher odds of malaria infection compared with their counterparts. This finding is consistent with previous studies [16, 31]. The higher odds observed among individuals engaged in outdoor activities may be associated with increased exposure to mosquito bites, particularly during night‐time when malaria vectors are often active [46]. However, the specific patterns of outdoor exposure and mosquito contact were not directly assessed in the present study. Furthermore, the odds of malaria parasite infection were 2.4 times higher among individuals who did not regularly wear long clothes at night compared with those who did. This result is in agreement with previous studies [42].

Those individuals who lacked knowledge about malaria prevention and control methods had two times higher odds of malaria infection compared with those who had such knowledge, and the association was statistically significant (*p* = 0.036). This finding agrees with previous studies [16, 42]. In addition, individuals who did not regularly clean their environment had two times higher odds of malaria infection compared with those who maintained environmental cleanliness, and this association was statistically significant (*p* = 0.032). This finding is in agreement with previous research [47]. Poor environmental sanitation may be associated with conditions that favor malaria transmission; however, the present study did not directly evaluate vector breeding habitats or mosquito density. Although the association between malaria infection and the presence of holes in house walls was not statistically significant, individuals living in houses with wall openings had approximately 1.8 times higher odds of malaria infection compared with those living in houses without wall openings. Furthermore, individuals with a history of previous malaria infection had twice the odds of malaria infection compared with those without previous infection. This association may reflect repeated exposure among individuals living in areas with higher malaria transmission risk rather than a causal effect of previous infection itself. This finding is supported by previous studies [31, 40, 48].

The present study was limited by the absence of entomological assessments, including *Anopheles* larval and adult mosquito surveys, vector density estimation, infection rate determination, human blood index estimation, and other vector‐related indicators. Therefore, future studies integrating human, parasite, and vector components are needed to provide a comprehensive understanding of malaria transmission dynamics in the study area.

## 5. Conclusion

This finding showed that although vector control strategies such as ITN use were implemented in the study area, the prevalence of malaria was 21.9%, indicating that malaria remains an important public health concern. All of the malaria cases detected in the study area were due to *P. falciparum*, *P. vivax,* and mixed infections, with relative proportions of 69.9%, 23.7%, and 6.5%, respectively. Age, residence, occupation, monthly family income level, presence of stagnant water near the home, knowledge of malaria prevention and control methods, environmental sanitation status, and ITN use were identified as factors associated with malaria parasite infection. Therefore, strengthening existing malaria prevention and control measures, including consistent IRS utilization, environmental management, health education, and timely diagnosis and treatment, is recommended. Further entomological and longitudinal studies are needed to evaluate malaria transmission dynamics and guide appropriate vector control interventions in the study area.

## Author Contributions

Abrhi Tsehay contributes to the study concept, data collection, analysis, and interpretation of the results. Sissay Menkir and Habtamu Mekonnen reorganized the study design, reviewed, and edited the overall manuscript.

## Funding

This study was supported by internal institutional funding from the Postgraduate Research and Community Service Office, College of Science, Bahir Dar University, Ethiopia (Grant No. SCI/0436/2014).

## Disclosure

The final manuscript was read and approved by all authors. This manuscript is based on the thesis entitled “Prevalence of malaria and its associated risk factors among malaria suspected patients attending Fenote Selam General Hospital, West Gojjam, North West Ethiopia” submitted to Bahir Dar University and available through the Bahir Dar University Institutional Repository (https://ir.bdu.edu.et/handle/123456789/14188) [49].

## Ethics Statement

The study protocol was ethically approved by the College of Science, Bahir Dar University (Ref. No. PRCSVD/294/2014), and permission was obtained from the Amhara Public Health Institute. The study objectives, risks, and benefits were explained to participants or their parents/guardians, and written informed consent was obtained. Participants who tested positive for *Plasmodium* species were referred for appropriate medical treatment.

## Conflicts of Interest

The authors declare no conflicts of interest.

## Data Availability

The data can be obtained from the corresponding author upon reasonable request.
